# Seasonal Dynamics and Metagenomic Characterization of Marine Viruses in Goseong Bay, Korea

**DOI:** 10.1371/journal.pone.0169841

**Published:** 2017-01-25

**Authors:** Jinik Hwang, So Yun Park, Mirye Park, Sukchan Lee, Taek-Kyun Lee

**Affiliations:** 1 South Sea Environment Research Department, Korea Institute of Ocean Science and Technology, Geoje, Republic of Korea; 2 Marine Environmental Science, Korea University of Science and Technology, Daejeon, Republic of Korea; 3 Department of Genetic Engineering, Sungkyunkwan University, Suwon, Republic of Korea; Oklahoma State University, UNITED STATES

## Abstract

Viruses are the most abundant biological entities in the oceans, and account for a significant amount of the genetic diversity of marine ecosystems. However, there is little detailed information about the biodiversity of viruses in marine environments. Rapid advances in metagenomics have enabled the identification of previously unknown marine viruses. We performed metagenomic profiling of seawater samples collected at 6 sites in Goseong Bay (South Sea, Korea) during the spring, summer, autumn, and winter of 2014. The results indicated the presence of highly diverse virus communities. The DNA libraries from samples collected during four seasons were sequenced using Illumina HiSeq 2000. The number of viral reads was 136,850 during March, 70,651 during June, 66,165 during September, and 111,778 during December. Species identification indicated that *Pelagibacter phage* HTVC010P, *Ostreococcus lucimarinus* OIV5 and OIV1, and *Roseobacter phage* SIO1 were the most common species in all samples. For viruses with at least 10 reads, there were 204 species during March, 189 during June, 170 during September, and 173 during December. Analysis of virus families indicated that the *Myoviridae* was the most common during all four seasons, and viruses in the *Polyomaviridae* were only present during March. Viruses in the *Iridoviridae* were only present during three seasons. Additionally, viruses in the *Iridoviridae*, *Herpesviridae*, and *Poxviridae*, which may affect fish and marine animals, appeared during different seasons. These results suggest that seasonal changes in temperature contribute to the dynamic structure of the viral community in the study area. The information presented here will be useful for comparative analyses with other marine viral communities.

## Introduction

Over the past two decades, studies of marine viruses with electron and fluorescence microscopy have revealed an unexpected abundance of virus particles, at 10^6^ to10^9^ particles per mL of seawater [1, 2]. Thus, viruses are the most abundant microbes in the sea, and most likely in the entire biosphere [3]. Although viruses account for approximately 5% of the total biomass in the ocean, they account for approximately 94% of all nucleic acid-containing particles [4, 5]. Therefore, marine viruses are regarded as potential pathogens that could infect large numbers of organisms and cause mass mortality in the oceans [6].

Metagenomics provides an in-depth characterization of the molecular diversity of DNA viruses in a range of environments, including marine ecosystems [7, 8]. Advances in next generation sequencing and sequence assembly techniques provide metagenomics studies with access to large genomic fragments rather than short reads, a previous limitation. These large assembled sequences provide access to the genome content and structure of uncultured viruses, and thereby allow unique insights into the main viral families in the environment [9]. Viral metagenomics studies extract nucleic acids directly from viral particle-enriched environmental samples, analyze these samples by next-generation sequencing [10], and then use bioinformatics to analyze the numerous sequences. One of the difficulties of marine viral metagenomics is the enrichment of viral particles from environmental samples. The flocculation, filtration, and re-suspension (FFR) method using FeCl_3_, which can enrich large volumes of marine viruses quickly and without expensive equipment, is frequently used to concentrate and extract marine viruses [11].

The viral community composition change depends on regional and local processes, including species interactions and environmental factors. Viral community changes can occur seasonally in response to variations in environmental parameters including temperature, salinity, dissolved oxygen, and chlorophyll *a*. In recent studies, the effect of environmental factors on viral communities has focused on the importance of temperature and nutrient concentrations [12, 13].

Research has focused on these variables because temperature can have a strong effect on biological processes and nutrient availability can drive niche structure through resource partitioning. A finding of particular importance to the present study is the recent demonstration that marine viruses have a latitudinal diversity gradient, with maximum richness at higher temperatures. However, many other factors modulate this effect. Given the dominance of bacteria in communities and ecosystem processes, prediction of ecosystem response to environmental changes requires assessment of the diversity of bacterial populations. Several studies demonstrated that it is possible to predict the taxonomic composition of viral communities from environmental parameters [14, 15].

Several factors, including climate change and influx of contaminants, have led to viral-associated diseases in ocean ecosystems in Korea. Emerging marine viral pathogens can have significant consequences for the production of many marine products, including fish, shellfish, and seaweed. In this study, we examined the distribution and abundance of marine viruses and their changes with the seasons in Goseong Bay, an important location for the aquaculture industry in Korea. The results of this study will contribute to our understanding of marine viral biogeography and provide a baseline for further studies of potential viral pathogens that may threaten aquaculture in Korea. This study of seasonal changes in viral composition is the first comprehensive study of viral populations in Goseong Bay.

## Materials and Methods

### Ethics statement

No specific permits were required for the described field studies. The location is not privately owned or protected in any way, and the field studies did not involve endangered or protected species.

### Sample collection

**Fig 1:**
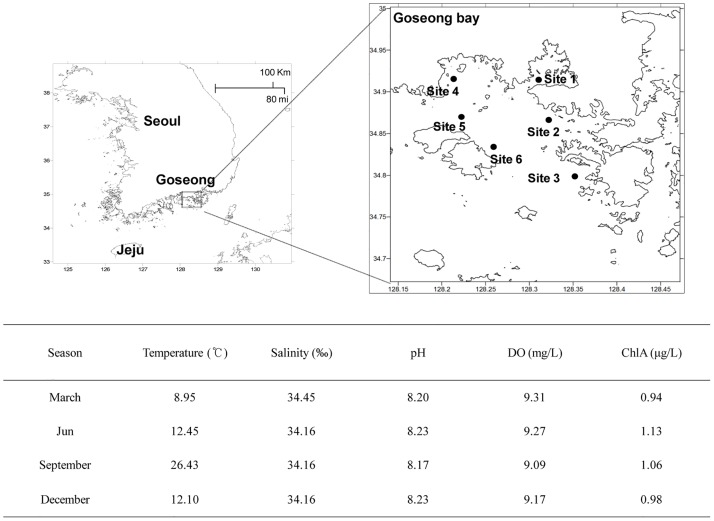
Location of sample sites (top) and environmental indices at these sites (bottom) in Goseong Bay, Korea. Temperature, salinity, pH, DO, and chlorophyll *a* were measured with a YSI 6600 Sonode.

Seawater was collected from 6 sites in the South Sea of Korea on 10 March, 6 June, 18 September, and 24 December in 2014. Each sample (30 L) was collected in sterile plastic bottles. We measured the basic biological characteristics of the seawater at these sites, including temperature, salinity, pH, dissolved oxygen, and chlorophyll *a* (Fig 1).

### Isolation of viral community DNA

The seawater samples were initially passed through a 3 μm filter (3M paper), and then a 0.22 μm filter (polycarbonate membrane, Millipore, USA) to remove debris and bacteria. For enrichment of marine viruses, we used FeCl_3_-mediated flocculation, as described previously [11, 16]. In brief, we added 6 mL of 1% aqueous FeCl_3_ into 60 mL of seawater and mixed it thoroughly. The sample was then incubated at room temperature for at least 1 h. The incubated seawater was then passed through a polycarbonate membrane filter (0.8 μm, Millipore, Billerica, MA, USA) and stored at 4°C until analysis. The filtered membranes, including viral particles and microbial cells, were cut into several pieces for DNA extraction. Total DNA was extracted using the IQeasy^™^ plus Viral DNA/RNA Extraction Kit (iNtRON Biotechnology, Seongnam, Korea) according to the manufacturer’s instructions. The DNA library for next generation sequencing (NGS) was prepared using the TruSeq Nano DNA library preparation kit (Illumina, San Diego, USA). The sequencing library was prepared by random fragmentation of the DNA or cDNA sample, followed by 5’ and 3’ adapter ligation. Alternatively, “tagmentation”, which combines the fragmentation and ligation reactions into a single step and greatly increases the efficiency of library preparation, was used. Adapter-ligated fragments were then amplified using PCR and purified by gel electrophoresis. The DNA library was pair-end sequenced using Illumina HiSeq 2000 by Macrogen (Seoul, Korea).

### Sequence analysis

Illumina SBS technology utilizes a proprietary reversible terminator-based method that detects single bases as they are incorporated into DNA template strands. Because all 4 reversible, terminator-bound dNTPs are present during each sequencing cycle, natural competition minimizes incorporation bias and greatly reduces raw error rates compared to other technologies. This method is a highly accurate base-by-base sequencing method that virtually eliminates context-specific errors in sequences, even within repetitive sequence regions and homopolymers. Raw data was blasted with a MegaBLAST that a parallel and fast nucleotide database search tool. In brief, two paired-end-sequenced FASTQ files were converted into FASTA using the FASTX-Toolkit (http://hannonlab.cshl.edu/fastx_too lkit/) and blasted against viral reference sequences (NCBI-BLAST, nucleotide) using default parameters that matched a NCBI viral reference 2.1.1 by BLAST with an E value 10^−3^.

### Classification of identified marine viruses

To classify the identified viruses according to viral taxonomy, the identified reference viral genome sequences were blasted against the viral reference genome sequences. The obtained XML file was imported into the MEGAN5 program [17]. The visualization of viral taxonomy and compared with viral reference sequences using default parameters.

## Results

We used the FASTX-Toolkit (http://hannonlab.cshl.edu/fastx_too lkit) with associated taxonomic browsing and filtering capabilities to explore the viral subset of annotations of metagenomic samples collected during March, June, September, and December from Goseong Bay, Korea. The resulting taxonomic comparison of these samples shows the relative proportions of different marine viruses

### Environmental characteristics

The salinity ranged from 34.16~34.45‰, the pH from 8.17~8.23, the DO from 9.09~9.31 mg/L, and the chlorophyll *a* from 0.94~1.13 μg/L throughout the year, with no significant seasonal variations (Fig 1). However, there was a marked seasonality of water surface temperature (8.95°C on March 10, 12.45°C on June 6, 26.43°C on September 18, and 12.10°C on December 24).

### Taxonomic composition

**Fig 2:**
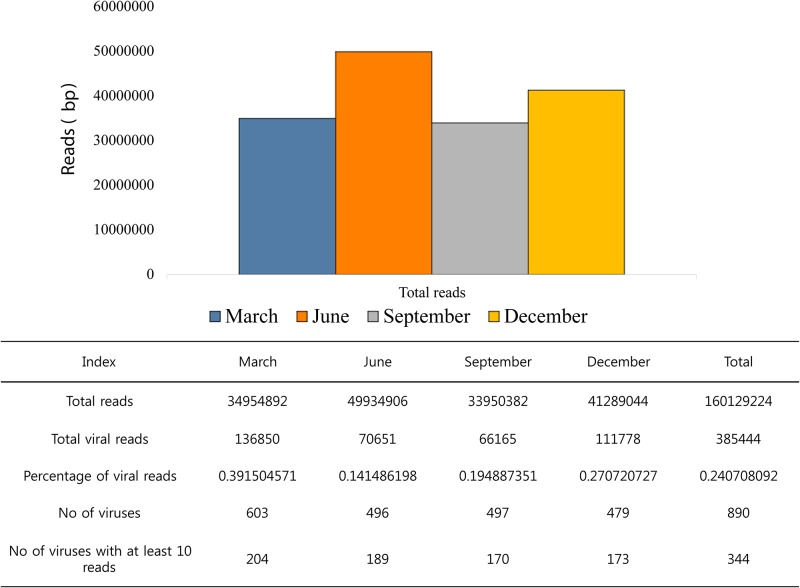
Number of total reads, total viral reads, fraction of reads that were viral, virus species, and virus species with at least 10 reads during each of the four seasons.

**Fig 3:**
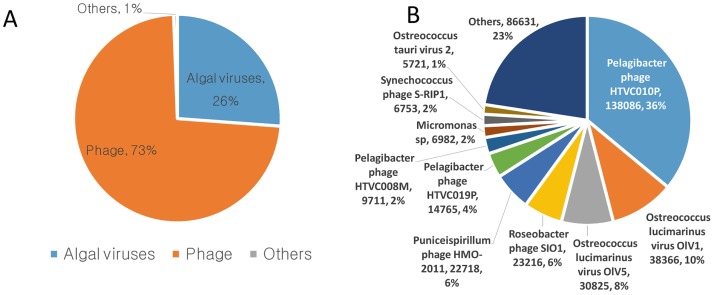
Abundance of different types of viruses based on read number (A) and abundance of different species based on read number (B).

**Fig 4:**
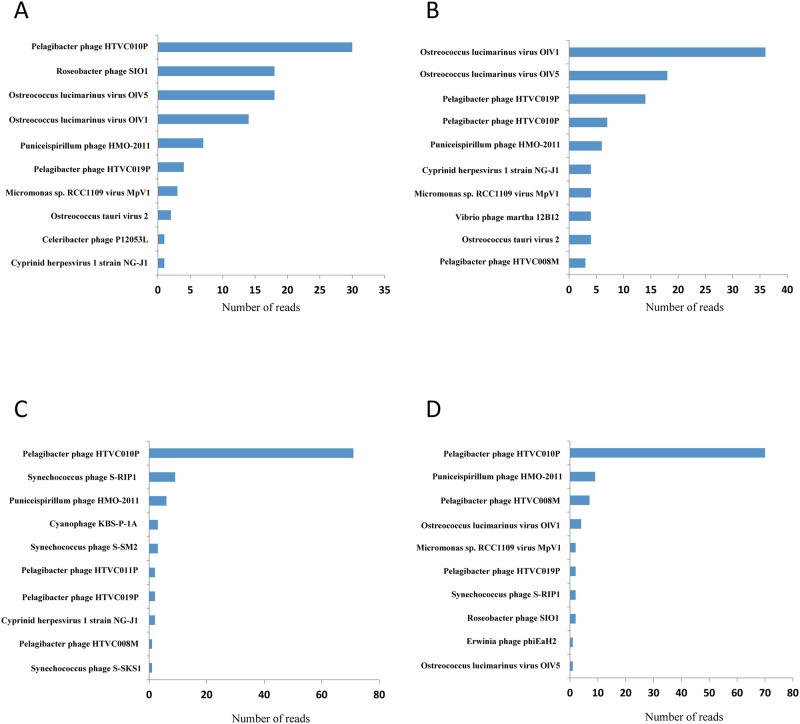
Abundance of the 10 most common virus species (based on read number) during March (A), June (B), September (C), and December (D).

To identify marine viruses, we used FASTX-Toolkit of DNA sequences followed by a BLAST search against the NCBI viral reference genome database. The numbers of viral reads in March, June, September and December in Goseong Bay were 136850, 70651, 66165 and 111778 reads, respectively (Fig 2). Total reads of four seasons were 385444, 73% of the sequences with significant hits were bacteriophage, and 26% were algal viruses and 1% were other viruses (Fig 3A). Species-level analysis indicated that *Pelagibacter phage* HTVC010P (36%) was the most abundant species, followed by *Ostreococcus lucimarinus* virus OIV5 (8%) and *Roseobacter phage* SlO1 (6%) (Fig 3B). Analysis of the most common viruses during different seasons indicated that *Pelagibacter phage* HTVC010P was most common in March (30%), September (71%) and December (70%), and *Ostreococcus lucimarinus virus* OlV1, OlV5 was most common in June (17, 26%) (Fig 4).

We obtained a large hit of reads matched to top 10 viruses. Therefore, it is likely that the obtained sequences can nearly cover the complete genomes. For this, we aligned all raw data on top 10 virus genomes in all seasons. As shown, most regions of viral genomes were covered by sequenced reads and some of viral genome regions were highly mapped (S1 Table).

**Fig 5:**
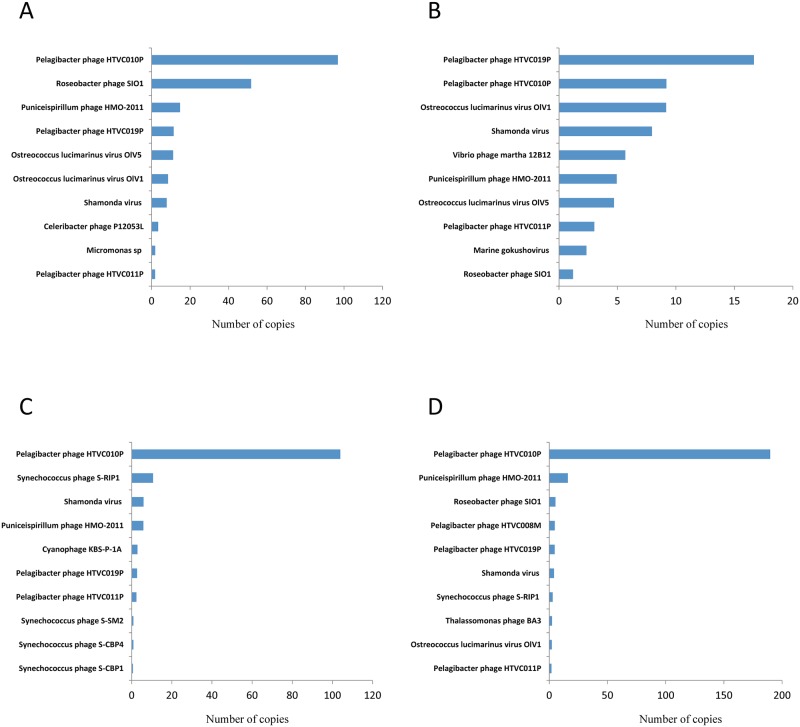
Abundance of the 10 most common virus species (based on copy number) during March (A), June (B), September (C), and December (D).

In general, the hit of identified viral sequences correlated with the size of the virus genome. Thus, we calculated the relative amount of each viral copy. *Pelagibacter* phage HTVC010P was again dominant in all seasons; however *Ostreococcus lucimarinus viruses* showed a lower copy than *Pelagibacter* phage. In general, viruses infecting algae have relatively large genome sizes; therefore, the DNA copy numbers were very low. For instance, the total viral copy number for three algae viruses OlV1 and OIV5 constituted only f 15 viral copies (Fig 5).

**Fig 6:**
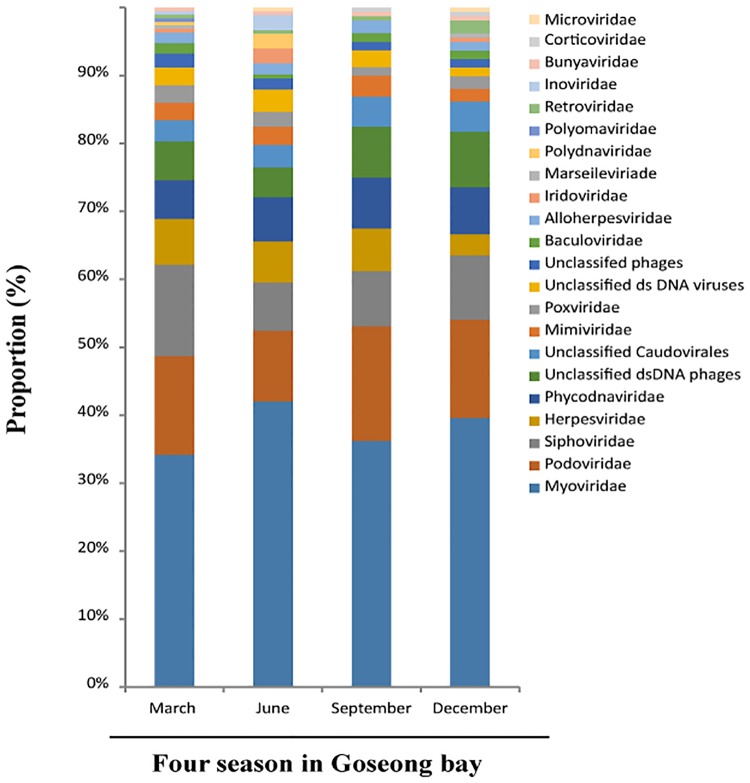
Relative abundance of viruses (based on read number) from different families, according to tBLASTn and the NCBI viral genome reference database (version 2.1.1) during each season.

Family-level analysis indicated that the viral communities were similar in the different seasons (Fig 5). In particular, viruses from the *Myoviridae* were the most common, followed by those from the *Podoviridae* and *Siphoviridae* in all seasons. *Myoviridae* accounted for the highest distributions in June, *podoviridae* won a number of distribution in September. In addition, *Baculoviridae* and *Streptococcaceae* were only detected in December and *Iridoviridae* was not detected in September (Fig 6).

**Fig 7:**
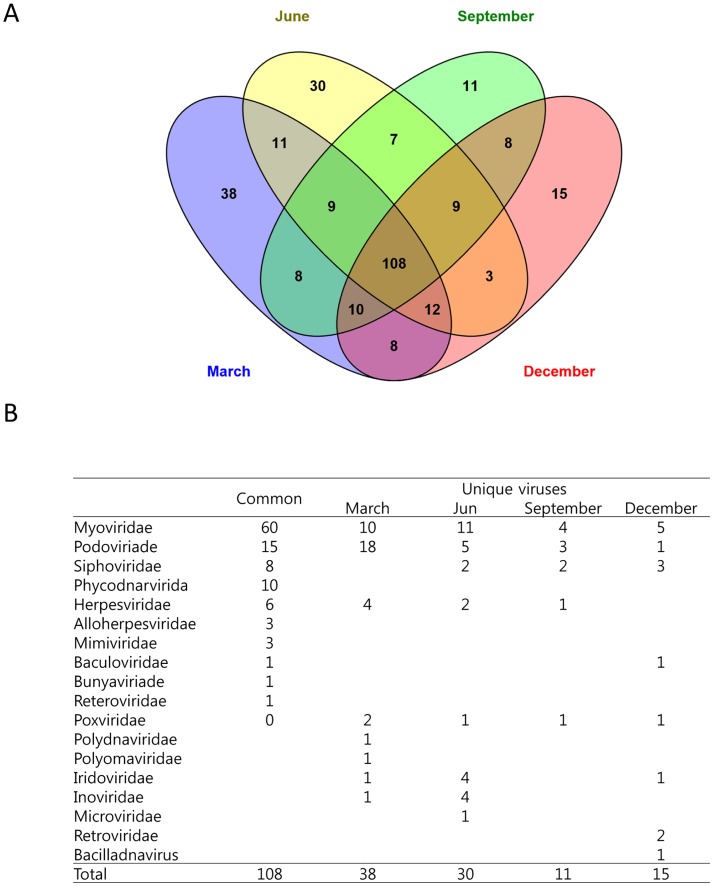
Presence of unique and common viruses from different families during different seasons (A) and number of virus species in different families (B).

Further investigation showed that 108 species hits were present in samples from all four seasons (Fig 7 and S2 Table). There were 38 unique species hits in March, 30 in June, 11 in September, and 15 in December. Among the 108 species hits present during all seasons, 60 were from the Myoviridae, 15 were from the *Podoviridae*, and 10 were from the *Phycodnaviridae* (Fig 7B). Among the unique seasonal viruses, viral abundance was higher in March than other seasons, and most of the unique species hits were from the *Podoviridae*. The numbers of *Iridoviridae* and *Inoviridae* species hits were greater during June than other seasons.

### Seasonal change of marine viruses

**Fig 8:**
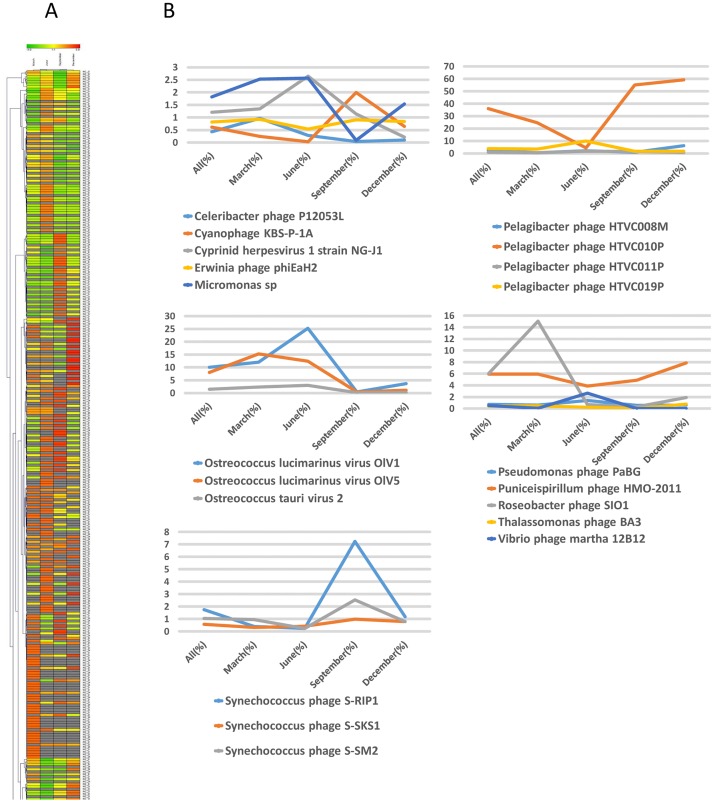
Relative abundance of different viral sequences at the species level during different seasons (A). The color code indicates abundance relative to the mean, and ranges from red (negative) to black (mean) to green (positive). Representative species that had significant seasonal changes (B).

Heat map analysis of the viral communities at the species level indicated that the dominant species changed with the seasons (Fig 8). For example, *Celeribacter phage* P12053L and *Cyprinid herpesvirus* 1 strain NG-J1 were more common during June, but less common during September. *Pelagibacter phage* HTVC010P was common in all samples except during June. *Ostreococcus lucimarinus virus* OIV1 and OIV5 were more common during March and June than other seasons. In addition, there were more sequences associated with *Roseobacter phage* SIO1 and *Synechococcus phage* S-RIP1 during March and September, respectively.

### Relationships between viral community and environmental factors

We measured the five environmental factors including temperature, salinity, pH, DO, Chla. Among the environmental indices we measured, temperature had the greatest seasonal variation (Fig 1). *Pelagibacter phage* HTVC010P and *Synechococcus phage* S-RIP1 were dominant during September when water temperature was highest; *Roseobacter phage* SIO1 was dominant during March when water temperature was lowest (Fig 8).

### Classification of identified marine viruses

We examined the taxonomy of identified viruses using the MEGAN5 program, which is frequently used for various metagenomic studies [17]. In March, the 204 viruses (excluding 8 unassigned viruses) were from 11 orders, 20 families, 51 genera, and 196 species (S1 Fig). In June, the 189 identified viruses (excluding 6 unassigned viruses) were from 11 orders, 18 families, 46 genera, and 183 species (S2 Fig). In September, the 170 identified viruses (excluding 3 unassigned viruses) were from 11 orders, 16 families, 41 genera, and 167 species (S3 Fig). In June, the 173 identified viruses (excluding 4 unassigned viruses) were from 11 orders, 19 families, 42 genera, and 169 species (S4 Fig).

## Discussion

Marine viruses are now recognized as abundant, diverse, and biogeochemically important members of marine ecosystems. For example, marine virus-like particles are highly abundant in plankton, and viruses can infect marine microorganisms and cause lysis. They can also influence elemental cycling, affect community diversity, and facilitate gene exchange among taxa [4, 18, 19]. In spite of their importance, there have only been limited studies of the marine virome due to technical challenges, insufficient bioinformatics on marine viruses, and limited analytical resources. Here, we report the use of metagenomics to identify seasonal marine viral communities in Korea. Metagenomics bridges the gap from genomes to ecology by identification of important genes and viral species, and by showing how viral communities behave in the context of their environments [20, 21]. Viral metagenomics also provides targets for molecular quantification of viruses in individuals [22] and their prevalence in populations [23], and for their distribution in the environment [24, 25].

Bacteriophages are the most abundant organisms in the biosphere and they occur in freshwater, soil, sewage, and marine environments [3, 26]. The high abundance of bacteriophages in the environment suggests they play important roles in ecology and evolution of diverse ecosystems [4]. Viruses also have enormous impacts on the global carbon cycle, and can thereby affect global climate. In particular, the marine ecosystem has numerous bacteriophages that play important roles, not only as predators of bacteria, but also in a wide range of ecological processes[27, 28]. The dominance of bacteriophages in our study of Goseong Bay means they play important roles in this marine ecosystem. A previous review of metagenomics research in Korea [29] reported that the *Microviridae* family was dominant. In contrast, we found that *Myoviridae* was the dominant virus family in Goseong Bay (Fig 5). The most abundant virus in our study was *Pelagibacter phage*, and we identified four types of this phage: HTVC010P, HTVC011P, HTVC019P, and HTVC008M. This result means that *Pelagibacter* ubique (the host, also called SAR11) must also be common in Goseong Bay (Fig 4). These bacteria feed on dissolved organic carbon and nitrogen in marine ecosystems. They are unable to fix carbon or nitrogen, but can perform the TCA cycle with the glyoxylate bypass and can synthesize all amino-acids. They also have an unusual and unexpected requirement for reduced sulfur [30, 31]. Infection of *Pelagibacter* by phages can cause a sharp decline in host numbers and the production of abundant phage particles. These relationships are clearly important for the marine ecosystem of Goseong Bay.

The second most common virus in Goseong Bay is *Ostreococcus lucimarinus virus* (OlV), and we identified two forms: OlV1 and OlV5. *Ostreococcus lucimarinus* is a *Prasinovirus* that infects algal plankton. Plankton are the smallest free-living eukaryotes known, and viral infection has an important role in microbial mortality [32–34]. In particular, they may shape the structure of communities by “killing the winner” [35] (*i*.*e*. elimination of the most abundant microbes, allowing rare and less competitive species to coexist), and may also drive the evolution of their hosts *via* horizontal gene transfer and promote an evolutionary “arms race”.

Interestingly, we also identified *Iridoviridae*, *Herpesviridae and Poxoviridae* in Goseong Bay (Fig 5). Iridoviruses infect marine organisms and can disturb marine ecosystems. Iridoviruses are large double-stranded DNA viruses which infect invertebrates and vertebrates, such as fish, insect, reptiles and mollusks. The mortality rates due to these iridovirus infections range from 30% to 100%. Iridoviruses have been isolated and identified from at least 20 fish species worldwide[36]. In particular, Red seabream iridovirus (RSIV) has caused significant economic losses in aquaculture. RSIV is a lethal pathogen of fish that major cause of mass fish mortality in several Asian countries. This viral disease has spread to more than 31 cultured marine fish species and has caused substantial economic losses in Japan [37]. RSIV has recently been classified in the new genus *Megalocytivirus*, along with the infectious spleen and kidney necrosis virus (ISKNV) [38] and the turbot reddish body iridovirus (TRBIV) [39].

Herpesvirus infections can cause disease in marine species, including Mollusc, bivalves, gastropod. These infections associated with high mortality rates have caused significant economic losses in aquaculture industry [40]. In particular, Ostreid herpesvirus 1 (OsHV-1) is one of the major pathogens affecting the oyster. OsHV-1 is assigned to the *Malacoherpes viridae* family and the *Herpesvirales* order [41, 42]. Over the past few decades, numerous studies of mortality outbreaks related to OsHV-1 in oysters have been reported worldwide, from Asia to Europe [43, 44].

*Poxvirus* infections of marine mammals are mainly evident through overt lesions. For example, virus infections of pinnipeds (seals) lead to the formation of cutaneous and occasionally oral nodules, and the responsible *poxvirus* almost always has the morphological characteristics of the *Parapoxvirus* genus [45, 46]. Species in this genus cause proliferative skin diseases in marine animals and humans [47]. Thus, our identification of *Iridovirus*, *Herpesvirus*, and *Poxviridae* in Goseong Bay indicates a potential or ongoing threat to the marine mammals and the seafood industry in this region.

Metagenomic analysis has become an invaluable tool for characterization of viral taxonomic composition and diversity from environmental samples. However, the procedures used for sample collection, sequencing preparation, and bioinformatics analysis are not all well established, and may induce biases in estimating viral diversity. In particular, due to the small size and biomass of viruses, enrichment steps are necessary to obtain sufficient experimental materials. Most other published studies of viromes used a 0.22 μm tangential flow filtration (TFF) step to remove cells. However, it is likely that this process removes large viruses as well, and this could bias the composition of the virome. Hence, we used a new chemistry-based concentration method—FeCl_3_ precipitation—that provides nearly complete recovery of viruses. Most previous research on aquatic viromes reported a high prevalence of *Caudovirales* sequences, although the Tara Oceans Expedition reported that non-tailed viruses were numerically dominant in the upper oceans [48].

Our study characterized the seasonal distribution of marine viruses in Goseong Bay. The results showed remarkable seasonal changes in 7 species: *Celeribacter phage* P12053L, *Cyprinid herpesvirus* 1 strain NG-J1, *Pelagibacter phage* HTVC010P, *Ostreococcus lucimarinus* OlV1, OlV5, *Roseobacter phage* SlO1, and *Synechococcus phage* S-RIP1. Our results suggest temperature has an important effect on the abundance of marine viruses and their hosts. Marine viruses can affect host growth rate and population size [49, 50]. Thus, future studies should investigate the correlation between marine viruses and their hosts. Our study of the composition and diversity of marine viruses in Goseong Bay will be useful as a foundation for comparative analyses of other marine viral populations.

## Supporting Information

S1 Fig Phylogenetic tree of viruses identified in March. Viral metagenomes using MEGAN. (TIF)

S2 Fig Phylogenetic tree of viruses identified in June. Viral metagenomes using MEGAN. (TIF)

S3 Fig Phylogenetic tree viruses identified in September. Viral metagenomes using MEGAN. (TIF)

S4 Fig Phylogenetic tree viruses identified in December. Viral metagenomes using MEGAN. (TIF)

S1 Table Identification of top 10 viral genome in all seasons. (XLSX)

S2 Table Common and unique virus groups in different seasons. (XLSX)
