# Supplementary figures and images for: Seasonal Dynamics and Metagenomic Characterization of Marine Viruses in Goseong Bay, Korea
Source: PLoS One. 2017 Jan 25;12(1):e0169841. doi: 10.1371/journal.pone.0169841 (PMC5266330; doi:10.1371/journal.pone.0169841)

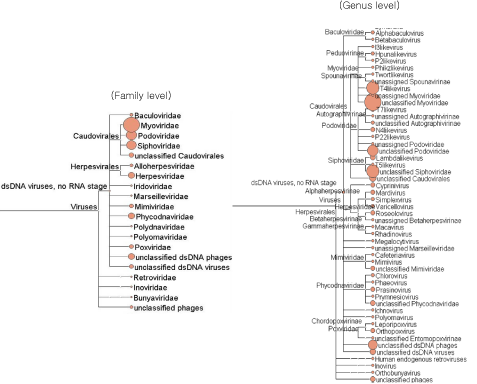

Supplement: S1 Fig — (TIF) [file pone.0169841.s001.tif]

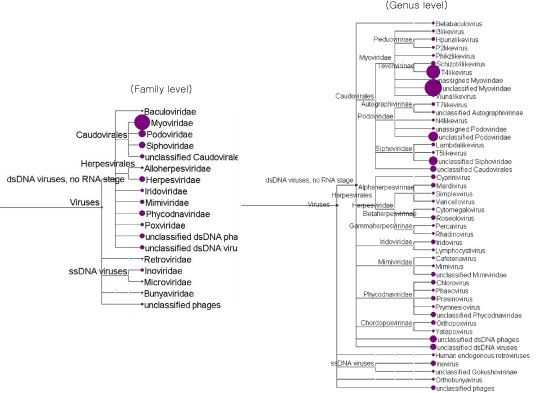

Supplement: S2 Fig — (TIF) [file pone.0169841.s002.tif]

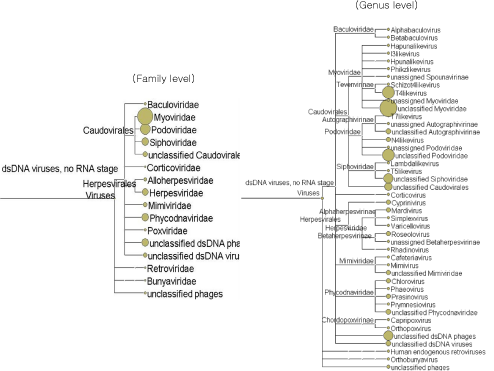

Supplement: S3 Fig — (TIF) [file pone.0169841.s003.tif]

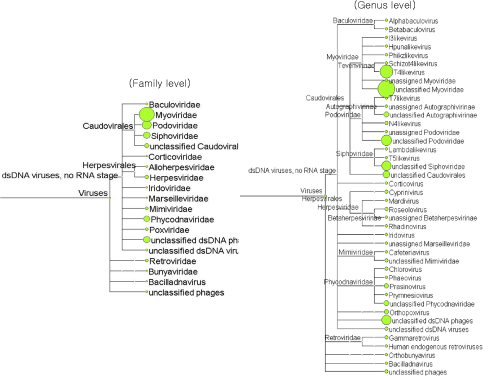

Supplement: S4 Fig — (TIF) [file pone.0169841.s004.tif]
